# A Pilot Randomized Controlled Trial of Goal Management Training in Canadian Military Members, Veterans, and Public Safety Personnel Experiencing Post-Traumatic Stress Symptoms

**DOI:** 10.3390/brainsci12030377

**Published:** 2022-03-12

**Authors:** Alina Protopopescu, Charlene O’Connor, Duncan Cameron, Jenna E. Boyd, Ruth A. Lanius, Margaret C. McKinnon

**Affiliations:** 1 Department of Psychology, Neuroscience and Behaviour, McMaster University, Hamilton, ON L8S 4L8, Canada; protopa@mcmaster.ca; 2 St. Joseph’s Healthcare Hamilton, Hamilton, ON L9C 0E3, Canada; henderds@mcmaster.ca (D.C.); boydj@stjosham.on.ca (J.E.B.); 3 Homewood Research Institute, Guelph, ON N1E 6K9, Canada; coconnor@homewoodhealth.com (C.O.); ruth.lanius@lhsc.on.ca (R.A.L.); 4 Homewood Health Centre, Guelph, ON N1E 6K9, Canada; 5 Department of Psychiatry and Behavioural Neurosciences, McMaster University, Hamilton, ON L8S 4L8, Canada; 6 Department of Psychiatry, Western University, London, ON N6C 0A7, Canada; 7 Imaging Division, Lawson Health Research Institute, London, ON N6C 2R5, Canada

**Keywords:** cognitive dysfunction, cognitive remediation, emotion regulation, goal management training, military, posttraumatic stress disorder, public safety personnel, veterans

## Abstract

Post-traumatic stress disorder (PTSD) is a severe psychiatric illness that disproportionately affects military personnel, veterans, and public safety personnel (PSP). Evidence demonstrates that PTSD is significantly associated with difficulties with emotion regulation (ER) and difficulties with cognitive functioning, including difficulties with attention, working memory, and executive functioning. A wide body of evidence suggests a dynamic interplay among cognitive dysfunction, difficulties with ER, and symptoms of PTSD, where numerous studies have identified overlapping patterns of alterations in activation among neuroanatomical regions and neural circuitry. Little work has examined interventions that may target these symptoms collectively. The primary objective of this pilot randomized controlled trial (RCT) with a parallel experimental design was to assess the effectiveness of goal management training (GMT), a cognitive remediation intervention, in reducing difficulties with cognitive functioning, and to determine its effects on PTSD symptoms and symptoms associated with PTSD, including difficulties with ER, dissociation, and functioning among military personnel, veterans, and PSP. Forty-two military personnel, veterans, and PSP between the ages of 18 and 70 with symptoms of PTSD were recruited across Ontario, Canada between October 2017 and August 2019. Participants were randomized to either the waitlist (WL) (*n* = 18) or the GMT (*n* = 22) condition. Participants in both conditions received self-report measures and a comprehensive neuropsychological assessment at baseline, post-intervention, and 3-month follow-up. Following their completion of the 3-month follow-up, participants in the WL condition were given the opportunity to participate in GMT. Assessors and participants were blind to intervention allocation during the initial assessment. A series of 2 (time) × 2 (group) ANOVAs were conducted to assess the differences between the WL and GMT conditions from pre- to post-intervention for the self-report and neuropsychological measures. The results demonstrated significant improvements in measures of executive functioning (e.g., verbal fluency, planning, impulsivity, cognitive shifting, and discrimination of targets) and trending improvements in short-term declarative memory for participants in the GMT condition. Participants in the GMT condition also demonstrated significant improvements from pre- to post-testing in measures of subjective cognition, functioning, PTSD symptom severity, difficulties with ER, dissociative symptom severity, and depression and anxiety symptoms. No adverse effects were reported as a result of participating in GMT. The results of this pilot RCT show promise that GMT may be a useful intervention to improve symptoms of cognitive dysfunction, symptoms of PTSD, and symptoms associated with PTSD within military personnel, veterans, and PSP. Future work is needed to address the small sample size and the durability of these findings.

## 1. Introduction

Post-traumatic stress disorder (PTSD) is a severe psychiatric illness that occurs as a result of experiencing, witnessing, or learning about a traumatic event or events [1]. Military personnel, veterans, and public safety personnel (PSP) (i.e., dispatchers, correctional workers, police officers, firefighters, paramedics, and emergency medical technicians) [2] are regularly exposed to potentially traumatic events, such as natural disasters, military conflicts, motor vehicle accidents, assaults, and death, due to the nature of their work [3], placing these individuals at increased risk for the development of PTSD relative to the general population [3,4,5]. Here, compared to Canadian civilians, where approximately 9% meet the criteria for lifetime prevalence of PTSD [6], the lifetime prevalence of PTSD within Canadian military personnel and veterans is estimated to be 22% [7]. Although no lifetime prevalence estimates of PTSD currently exist for PSP, approximately 23% of PSP met the diagnostic criteria of PTSD within the past month when assessed using a web-based self-report survey [3].

According to the *Diagnostic and Statistical Manual of Mental Disorders, Fifth Edition* (DSM-5) [1], PTSD is characterized by re-experiencing symptoms (e.g., intrusive and distressing memories, dreams, or flashbacks), avoidance, alterations in mood and thoughts (e.g., negative beliefs about oneself, others, the world; persistent low mood or anhedonia), and changes in arousal (e.g., hypervigilance, angry outbursts). Individuals with PTSD may also experience symptoms of dissociation associated with feelings of derealization (i.e., feeling as though the world around oneself is unreal or strange) or depersonalization (i.e., perception of the self feels unreal or strange) and may be diagnosed with the dissociative subtype of PTSD [1]. Notably, approximately 16% to 19% of veterans diagnosed with PTSD meet the criteria for this subtype [8].

PTSD is also comorbid with several other psychiatric disorders, including major depressive disorder (MDD) [1,6,9], anxiety disorders [1,9], and alcohol and substance use disorders [1,6,9]. For example, among a Canadian military personnel and veteran sample diagnosed with PTSD during their lifetime, approximately 80% also had a comorbid diagnosis of MDD, 59% had a comorbid diagnosis of social anxiety disorder, 51% had a comorbid diagnosis of panic disorder, and 46% had a comorbid diagnosis of generalized anxiety disorder [7].

Critically, PTSD is highly associated with difficulties with emotion regulation (ER), a transdiagnostic factor across various psychiatric conditions that refers to difficulties with moderating and managing emotions [10,11,12,13,14,15,16,17,18,19]. Here, elevated levels of difficulties with ER are associated with increased PTSD symptom severity and expression [13,14,16,18,19]. Moreover, symptoms of dissociation associated with PTSD may serve as a form of ER strategy, allowing individuals to disengage from experiencing highly distressing emotions [15]. Together, these findings suggest that difficulties with ER may contribute to PTSD symptom maintenance and point towards the need for targeted treatment approaches for the resolution of ER difficulties among individuals with PTSD [20]. Despite this knowledge, to date, systematic approaches for the treatment of ER difficulties are seldom employed in the treatment of PTSD, instead forming a relatively small component of more cognitively oriented approaches such as cognitive processing therapy [21,22] and prolonged exposure therapy [23]. 

Along with other neuropsychiatric presentations, such as MDD and anxiety disorders, PTSD is also associated with difficulties in cognitive functioning [1,24]. Here, negative alterations in verbal learning, declarative memory [25,26,27,28], attention, working memory, executive functioning [26,29,30,31,32], and processing speed [26,28] are routinely observed among individuals with PTSD. Data from a meta-analysis have demonstrated medium effect size impairments associated with PTSD occurring in verbal learning (Cohen’s *d* = −0.62), processing speed (Cohen’s *d* = −0.59), and attention and working memory (Cohen’s *d* = −0.50), with executive functioning (Cohen’s *d* = −0.45), verbal memory (Cohen’s *d* = −0.46), language (Cohen’s *d* = −0.43), visuospatial functioning (Cohen’s *d* = −0.38), visual learning (Cohen’s *d* = −0.32), and visual memory (Cohen’s *d* = −0.29) demonstrating smaller effect sizes [28]. Notably, this meta-analysis found an overall medium effect size impairment of PTSD on neurocognitive functioning (Cohen’s *d* = −0.49). In another meta-analysis, the researchers compared executive functioning in a PTSD group to healthy and trauma-exposed control groups [32]. Here, it was found that individuals in the PTSD group demonstrated small to moderate impairments in executive functioning (i.e., Hedges’ *g* = 0.464 and Hedges’ *g* = 0.414 for the healthy and trauma-exposed control groups, respectfully), independent of PTSD symptom severity, suggesting that difficulties with executive functioning may be present across varying levels of PTSD symptom severity. These findings also align with previous meta-analysis results [28]. 

Additional work has explored cognitive difficulties among individuals with PTSD and comorbid dissociative symptoms [33,34,35]. Here, it has been found that there are negative alterations in verbal and visual memory [34], attention, executive functioning, and autobiographical memory [35] among individuals with PTSD and co-occurring dissociative symptoms. In conjunction with these objective cognitive difficulties, individuals with PTSD also report subjective difficulties with cognitive functioning [36,37], which are associated with increased psychological symptom severity among military veterans [38] and civilians [39]. 

Cognitive dysfunction also is associated with negative impacts on physical [40], social, and occupational functioning (e.g., increased absenteeism) [40,41], as well as poorer psychological treatment outcomes, among military personnel and civilians with PTSD [42,43,44,45]. These difficulties may be long-standing, where alterations in cognitive performance continue to persist in approximately 25% of individuals who have completed either cognitive processing therapy or prolonged exposure therapy [46]. Like difficulties in ER, however, negative alterations in cognitive functioning (i.e., both objective and subjective difficulties) are rarely addressed as a specific treatment target in PTSD, despite their associations with poorer outcomes and increased symptom severity [36]. 

Importantly, a wide body of evidence suggests a dynamic interplay among cognitive dysfunction, difficulties with ER, and symptoms of PTSD, where numerous studies have identified overlapping patterns of alterations in activation among neuroanatomical regions [15,47,48,49,50,51,52,53] and large-scale neural circuitry [54,55,56,57]. Here, reductions in activation of pre-frontal cortical regions, such as the medial pre-frontal cortex and the rostral anterior cingulate cortex, have been associated with decreases in top-down control of emotion processing and regulation centres of the brain, such as the amygdala and other limbic regions [15,50,51]. Conversely, increases in activation of pre-frontal cortical regions have been associated with increases in top-down control of emotion processing and regulation centres of the brain. This profile of hypoactivation of pre-frontal cortical regions and concomitant hyperactivation of emotion processing and regulation centres of the brain is thought to contribute to emotional undermodulation and associated re-experiencing and hyperarousal symptoms among individuals with PTSD [15,50]. Comparatively, hyperactivation of pre-frontal cortical regions and concomitant hypoactivation of emotion processing and regulation centres of the brain contribute to emotional overmodulation, which is associated with symptoms of dissociation. Critically, many of these same pre-frontal cortical regions are associated with higher-order cognitive functioning, including executive functioning, working memory, and attentional control [58,59], with alterations in neural activity thought to underlie, in part, alterations in cognitive functioning among individuals with PTSD [26,29,30,31,32]. 

These neuroanatomical regions form larger brain networks, including the default mode network (DMN), the central executive network (CEN), and the salience network (SN) [60,61,62]. In the absence of any significant dysfunction, these neurocognitive networks work in conjunction with one another and are responsible for the top-down control of various processes, including cognitive functioning, emotions, and ER. Specifically, the DMN is associated with self-referential mental activity, such as self-monitoring, autobiographical memory, and future- or past-oriented thinking [61,63]. It is typically activated during rest and deactivated during cognitive tasks. Moreover, the DMN is anchored within the posterior cingulate cortex, the medial pre-frontal cortex, and the medial temporal lobe [61]. The CEN contributes to executive functioning, attention, memory, goal-directed behaviours, and the cognitive control of emotions and ER. It includes nodes in the pre-cuneus, dorsolateral pre-frontal cortex, and lateral posterior parietal cortex [60,61,64]. Finally, the SN is involved with directing attention to relevant internal and external stimuli and is co-activated with the CEN during cognitive tasks [60]. It is associated with nodes within the amygdala, anterior cingulate cortex, and anterior insula [60,61]. 

Like the evidence demonstrating differences in neural activity amongst the different brain regions in individuals with PTSD and its dissociative subtype [15,50], evidence demonstrates that these neural networks show aberrant patterns of connectivity at rest and during symptom provocation [56,65,66,67,68,69,70,71,72,73]. Generally, findings have shown patterns of hypoconnectivity within the DMN [55,74,75,76], hypoconnectivity between the DMN and the CEN [54,55,56,74,76,77], hypoconnectivity within the CEN [55,56,75], and hyperconnectivity within the SN [54,55,56]. Critically, these patterns of connectivity are associated with symptoms of PTSD, including symptoms of dissociation, avoidance, re-experiencing symptoms, a reduced ability to switch between task-relevant and task-irrelevant behaviours, cognitive deficits, hypervigilance, and fear [54,55,56,70]. Moreover, evidence has demonstrated differential patterns of neural connectivity among individuals with PTSD, individuals with the dissociative subtype of PTSD, and healthy controls [70]. For example, among individuals with the dissociative subtype of PTSD, Nicholson et al. (2020) reported that there were increased connections amongst nodes within the DMN, such as the middle dorsal pre-frontal cortex, as well as hyperconnectivity within the CEN, in comparison to individuals with PTSD alone. It was hypothesized that the hyperconnectivity levels within the DMN and CEN were associated with symptoms of emotional overmodulation and subsequently the presentation of dissociative symptoms, such as depersonalization and derealization [15,68,69,78]. Such patterns of connectivity within the DMN and CEN have been postulated to be associated with changes in cognitive functioning found amongst individuals with PTSD and co-occurring dissociative symptoms [33]. Among individuals with PTSD, the findings demonstrated hyperconnectivity between the posterior SN and the anterior insula relative to individuals with the dissociative subtype [70]. Notably, this pattern of hyperconnectivity between the posterior SN and the anterior insula within the PTSD group is thought to reflect increased internal and external stimuli processing [56,62,67], which can contribute to symptoms of hypervigilance and increased reactivity [79].

Disruption to these networks may confer difficulties with top-down cognitive control and bottom-up regulation, subsequently disrupting both cognitive function and ER [52,55,56,57]. In many instances, for example, the ability to successfully regulate emotions is dependent on the ability to inhibit a pre-potent response and executive control [80,81]. For example, cognitive ER, which is the ability to reappraise the meaning of an initial stimulus such that the emotional response is altered [82,83,84], is dependent upon cognitive processes such as working memory, executive functioning, and attention [82,83,85,86]. Notably, impairments in these cognitive functions have been associated with increased risk of developing psychiatric disorders, such as anxiety [87]. Conversely, ER is necessary for successful cognitive functioning, such that different ER strategies confer different cognitive benefits or consequences [80,88]. For example, in a study by Richards and Gross (2000), the researchers examined whether differences between the types of ER strategy impacted memory [88]. Here, it was found that individuals who utilized expressive suppression (i.e., inhibiting behavioural urges to act on emotions) performed significantly worse on tasks of nonverbal and verbal memory versus those who engaged in cognitive reappraisal during an emotion-inducing slide presentation.

Given the wide-ranging dysfunction associated with the loss of top-down cognitive control in PTSD, an intervention that assists with reinstating cognitive control and favors the implementation of generalizable skills to address multiple neurocognitive domains, such as impulsivity and executive functioning, may be of benefit. Specifically, such an intervention may not only address the cognitive dysfunction associated with PTSD but also symptoms associated with PTSD, such as difficulties with ER and dissociation, given the commonalities amongst the neuroanatomical regions and networks associated with these symptoms [56]. Such an intervention may include goal management training (GMT) [59,89], which is a skills-based, top-down, cognitive remediation intervention. It is theorized that GMT targets the brain’s sustained attention system [59,90,91,92]. This system is activated within the dorsolateral pre-frontal cortex, posterior parietal cortex, and thalamic regions of the brain [92], which are regions associated with executive functioning. Moreover, this network shares similar neuroanatomical regions with the CEN [60,61,64]. Critically, disruption to the sustained attention system can impede higher-order goal-directed behaviours, such that automatic responding or other behaviours inconsistent with these goals can subsequently supersede these goals [59]. This may contribute to difficulties with attention and executive functioning. The purpose of GMT is to assist with the reinstatement of executive and supervisory control, such that individuals are taught skills and strategies to monitor goals [59,93] and to subsequently interrupt automatic responding [59]. 

GMT has been used to addresses cognitive difficulties across a variety of medical and neuropsychiatric disorders, including traumatic brain injury (TBI) [59], attention deficit hyperactivity disorder (ADHD) [94], substance abuse [95], and spina bifida [96]. It consists of nine two-hour, group-based sessions that provide individuals with psychoeducation, self-monitoring, and mindfulness-based strategies, as well as other skills to reduce the frequency and severity of cognitive difficulties, including difficulties with planning, organizing, attention, and memory [89]. A recent meta-analysis found that across various medical (e.g., TBI, multiple sclerosis) and neuropsychiatric (e.g., ADHD, substance use disorders) conditions, there was a significant small to moderate positive effect of GMT on executive functioning (Hedges’ *g* = 0.227), which remained during follow up testing (Hedges’ *g* = 0.549) [97]. Moreover, the meta-analysis revealed significant small to moderate positive effects of GMT in the domains of working memory (Hedges’ *g* = 0.438), long-term memory (Hedges’ *g* = 0.269), instrumental activities of daily living (e.g., cleaning the house, managing finances, cooking) (Hedges’ *g* = 0.662), and various psychiatric symptoms (Hedges’ *g* = 0.309) [97].

Recent work has sought to apply GMT within psychiatric samples [98,99]. For example, work by Cameron et al., (2020) examined the feasibility of GMT within an obsessive–compulsive disorder outpatient sample [98]. Relative to waitlist controls, individuals in the GMT condition improved on neuropsychiatric measures assessing problem-solving, planning, impulsivity, attention, and processing speed. Individuals in the GMT condition also demonstrated significant improvements on subjective ratings of cognitive functioning, as well as on self-report measures of functioning, including lessened interference of their obsessive–compulsive disorder symptoms during daily functioning and improvements in instrumental tasks of daily living. Studies have also begun to directly examine PTSD and trauma-exposed samples. Specifically, Boyd et al. (2019) conducted an initial open-label feasibility trial of a modified GMT protocol within an inpatient trauma-exposed sample [99]. The results from this study demonstrated that patients who participated in a modified GMT protocol while receiving treatment as usual experienced improvements in several areas of cognitive functioning, including executive functioning, processing speed, sustained attention, and verbal memory. In addition, relative to patients who did not participate in GMT, patients receiving GMT as an adjunctive treatment also reported an improvement in their ability to engage in goal-directed behaviours while experiencing difficulties with ER. Together, these initial findings suggest that GMT may not only be effective in improving areas of cognitive dysfunction (i.e., both objective and subjective cognitive functioning) typically associated with PTSD, but also that GMT has the potential to improve symptoms associated with PTSD, such as difficulties with ER, dissociation, and difficulties with functioning. 

### Study Objectives and Hypotheses

To date, no randomized control trial of GMT has been conducted among individuals experiencing symptoms of PTSD, nor have its effects been fully elucidated within a military, veteran, and PSP sample expected to experience PTSD and its deleterious impacts at a higher prevalence than their civilian counterparts [3,4,5,100]. Moreover, as cognitive dysfunction can persist among individuals who have received treatment for PTSD [46], participants who did not meet a current diagnosis of PTSD (e.g., those who had a history of a PTSD diagnosis or who exhibited symptoms of PTSD that did not meet the full diagnostic criteria) were also considered for inclusion within the trial. Accordingly, the primary aim of the present study was to build upon Boyd et al.’s (2019) previous findings [99] and conduct a pilot randomized controlled trial (RCT) to determine the effectiveness of GMT as compared to a waitlist (WL) condition in a sample of military personnel, veterans, and PSP experiencing symptoms of PTSD. We hypothesized that individuals randomly assigned to receive GMT in comparison to the WL condition would experience improvements in areas of objective measures of cognitive functioning, including executive functioning, processing speed, sustained attention, and verbal memory, as well as in a measure of subjective cognitive functioning. In addition, we hypothesized that participants assigned to the GMT groups would show significantly greater functional improvement compared to participants in the WL condition. Finally, we predicted that participants in the GMT condition compared to those in the WL condition would experience a subsequent improvement in symptoms of PTSD and symptoms associated with PTSD, including difficulties with ER, dissociation, depression, and anxiety. 

In addition to these objectives and hypotheses, a secondary objective of the study was to explore the trajectory of symptom change (i.e., self-reported cognitive difficulties, PTSD symptoms, difficulties with ER, dissociative symptoms, depression symptoms, and anxiety symptoms) over the course of GMT. It was hypothesized that the severity of these self-reported symptoms would decrease over the course of GMT. Moreover, given the noted interactions among PTSD symptoms, symptoms associated with PTSD, and cognition, an additional aim was to explore whether any self-reported symptoms at baseline (e.g., self-reported cognitive difficulties, difficulties with ER, etc.) influenced the trajectory of symptom change across the GMT intervention. Given that these analyses were exploratory in nature, no hypotheses regarding these outcomes were made.

## 2. Materials and Methods

This study was approved by the Homewood Health Centre Regional Centre for Excellence in Ethics (REB 15–29) and is registered at ClinicalTrials.gov (NCT04076839). A copy of the study protocol is available upon request.

### 2.1. Participants

The participants were 42 men (64.3% of the sample) and women (35.7% of the sample) who were either current or former military personnel, veterans, or PSP. Individuals were recruited by a research coordinator (i.e., a paid staff member of the Homewood Research Institute (HRI)) during recruitment groups at the Homewood Health Centre (HHC) while completing treatment on the Program for Traumatic Stress Recovery (PTSR) inpatient unit in Guelph, Ontario, Canada. Additionally, recruitment occurred at HHC’s Outpatient clinic in Mississauga, Ontario, the PTSR’s external referral agencies (e.g., Military Family Resource Centres, Department of National Security Case Management), external community agencies and support groups (e.g., Military Causality Support Foundation, Project Trauma Support, etc.), and social media websites (e.g., Facebook groups, Twitter, etc.) via recruitment posters, information letters, e-mails, social media postings, and directly through clinician or staff member referrals at recruitment sites. 

To be included in the study, participants had to meet the following inclusion criteria: (a) be between the ages of 18 and 70; (b) have a diagnosis or a history of a PTSD diagnosis based upon the Clinician-Administered PTSD Scale for DSM-5 (CAPS-5) [101], meet or exceed the clinical cut-off score on the PTSD Scale for DSM-5 (PCL-5) [102], or have a history of trauma as indicated on the Life Events Checklist (LEC) [103]; (c) be fluent in written and spoken English; (d) be able to provide written informed consent; (e) have been discharged from the PTSR inpatient unit, if applicable. Exclusion criteria included: (a) individuals who had no history of employment as military personnel, veterans, or PSP; (b) individuals who had a history of alcohol or substance abuse within the past three months; (c) individuals who had a history of a medical disorder within the past year known to adversely affect cognition (e.g., severe TBI).

Participants were randomly allocated to either participate in the GMT group intervention (*n* = 22) or were assigned to the WL condition (*n* = 20). Within both conditions, one individual switched from GMT to the WL condition and from the WL condition to GMT due to other scheduling commitments, leaving the initial sample sizes the same. Within the GMT condition, one individual dropped out following their baseline assessment, while three individuals began the GMT group but dropped out of the group before completing the sessions. Here, 18 individuals attended and completed the GMT sessions (i.e., 83.3% of the GMT condition sample attended seven or more GMT sessions). All 18 individuals completed the GMT post-testing assessments. Fifteen individuals completed the three-month post-testing assessments. Three individuals did not complete their three-month post-testing appointments due to loss of contact. 

**Figure 1:**
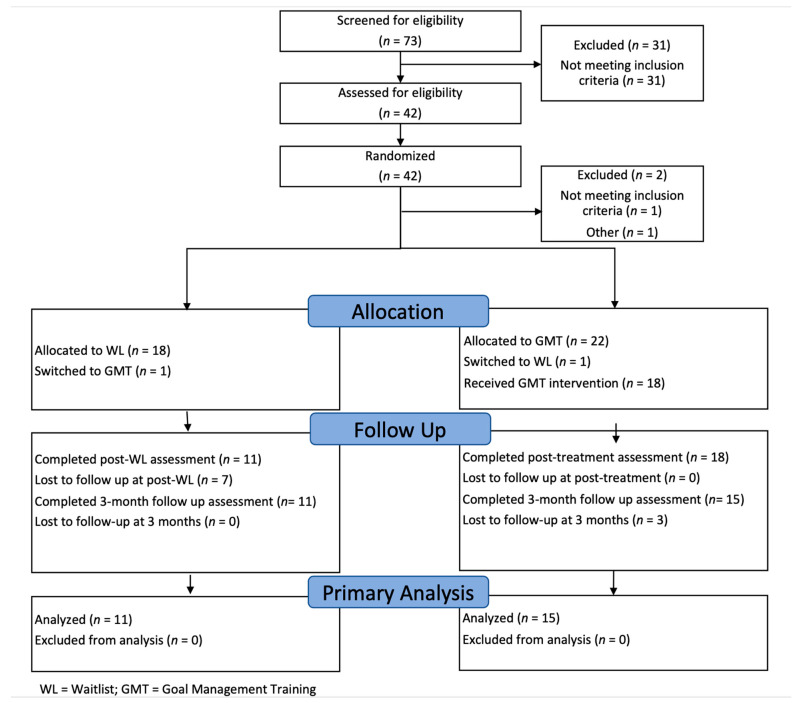
CONSORT diagram depicting recruitment, drop-out, and follow-up of study participants.

Within the WL condition, two individuals did not meet the criteria for the study during the baseline assessment and were excluded from the WL sample. Here, one participant was excluded as they were not a military personnel, veteran, or PSP, while the other participant identified that they had completed a previous GMT study with the same principal investigator. Of the 18 eligible individuals, 11 participants completed the post-delay testing. Eleven participants also completed the three-month delay post-testing assessment; however, one of these participants did not complete the initial post-delay assessment appointment. Therefore, only ten participants completed both the post-delay and three-month delay assessment appointments. Nine individuals from the WL condition attended and completed the GMT sessions (i.e., 88.9% attended seven or more GMT sessions). See Figure 1 for a CONSORT diagram depicting study recruitment, enrollment, and loss to follow up. Demographic and clinical characteristics of the initially eligible GMT and WL participants are reported in Table 1.

### 2.2. Experimental Design and Procedure

This study followed a pilot RCT with a parallel experimental design, which recruited and followed participants between October 2017 and August 2019. The website https://www.sealedenvelope.com/simple-randomiser/v1/lists (last accessed on 5 September 2017) was used to generate the random allocation sequence. Ten blocks were specified so that there would be an equal number of participants allocated to the GMT and WL conditions [104,105]. A randomization list was created for each site where participants were assessed and participated in GMT (i.e., HRI, Guelph, Ontario and HHC Outpatient Clinic, Mississauga, Ontario). The randomization lists were password protected and were only accessible by the research coordinator.

The research coordinator contacted participants who indicated their interest in the study and screened them for their eligibility. If eligible, the initial baseline testing appointment was scheduled with a trained assessor (i.e., a graduate student or individual with higher credentials and experience). Participants selected which research site they wished to attend before their initial assessment appointment. 

During the baseline assessment, the assessors and participants were blind to the intervention allocation. Following informed consent, baseline testing consisted of a battery of assessor-administered and self-report assessment measures that included psychological, functional, and neuropsychological assessments. The research coordinator contacted participants following the initial baseline assessment to inform them of their intervention allocation (i.e., GMT or WL). 

Individuals who were randomized to receive GMT participated in the 9-week, group-based cognitive remediation intervention. The GMT groups were closed, and each group consisted of four to ten participants to ensure adequate group facilitation and group member participation. During GMT, a battery of self-report measures was administered to participants at the initial GMT session and every third session thereafter. Following GMT, participants were assessed during a post-intervention assessment and again at a 3-month follow-up assessment. 

After their baseline assessment, participants who were assigned to the WL condition were assessed following a 9-week waiting period and at a 3-month follow-up. Following this 3-month assessment, participants were given the opportunity to participate in GMT. Those who participated in GMT were administered the same battery and schedule of self-report measures during the GMT intervention (i.e., initial GMT session and every third session thereafter). 

### 2.3. Study Conditions

GMT. As previously described, GMT consists of nine two-hour, weekly, group-based sessions that provide individuals with psychoeducation, self-monitoring strategies, mindfulness-based strategies, and other skills to reduce the frequency and severity of cognitive difficulties, including difficulties with planning, organizing, attention, and memory [89]. The GMT sessions were facilitated by an occupational therapist with significant GMT facilitation experience and treatment of military personnel, veterans, and PSPs with PTSD and trauma histories. Given this, GMT was tailored to this population by including psychoeducation and examples related to how PTSD and trauma can affect cognition (e.g., acknowledging how difficulties with dissociation, ER, and hypervigilance can affect attentional resources). These modifications to the protocol were not previously assessed and were also being piloted within this study. GMT also was co-facilitated by a clinical psychology graduate student who assisted with the group’s administration and session content, as needed. 

WL. Participants who were randomly assigned to the WL condition were required to wait for at least a three-month period after which they were offered the ability to participate in GMT. Participants completed testing following the initial nine-week waiting period, as well at the three-month follow-up. As previously described, following their participation in GMT (if elected), participants were assessed following the intervention and again at 3-months post-intervention. 

### 2.4. Measures

#### 2.4.1. Clinician-Administered Interviews

The *Mini International Neuropsychiatric Interview 7.0* (M.I.N.I.) [106] is a semi-structured, clinician-administered interview, which assesses 17 psychiatric disorders, including mood, anxiety, alcohol, and substance use disorders according to the DSM-5 [1]. The M.I.N.I. was only administered during baseline testing. 

The *Clinician-Administered PTSD Scale for DSM-5* (CAPS-5) [101] is a semi-structured interview that assesses the DSM-5’s PTSD diagnostic criteria in the past month. It assesses the onset, duration, frequency, and intensity of symptoms. The CAPS-5 was administered at baseline only. 

#### 2.4.2. Neuropsychological Assessment

A standardized neuropsychological assessment battery aimed at assessing intellectual functioning, executive functioning, processing speed, attention, and memory was administered to both GMT and WL condition participants. 

Intellectual functioning (assessed at baseline only): (a) the *Wechsler Test of Adult Reading* (WTAR) [107] was used to assess pre-morbid intellectual functioning in adults; (b) the *Wechsler Abbreviated Scale of Intelligence—Second Addition* (WASI-II) [108] is a brief estimate of intelligence. Here, the Vocabulary and Matrix Reasoning subtests were administered to yield a Full-Scale Intelligence Quotient (FSIQ). 

Measures of executive functioning, processing speed, and attention (administered at baseline, post-intervention, and at three months post-intervention for the GMT and WL conditions): (a) the *Controlled Oral Word Association Task* (COWAT) [109] was used to assess verbal fluency, including phonemic (FAS) and semantic (animals) fluency; (b) the *Stroop Color and Word Test* [110,111] was used to assess processing speed (word and color reading) and sensitivity to suppress habitual responses; (c) the *Delis-Kaplan Executive Function System* (DKEFS) *Tower Test* [112] was used as a measure of planning, rule learning, response inhibition, and perseveration; (d) the *Wechsler Adult Intelligence Scale-IV* (WAIS-IV) *Digit Symbol Coding Subtest* [113] assessed processing speed in adults; (e) the *Trail Making Test* (TMT) *Part A and B* [109] was used as a measure of attention, speed, and mental flexibility. Participants were required to connect numbers in sequential order (TMT part A) and numbers and letters in alternating order (TMT part B) as quickly as possible; (f) *Conners’ Continuous Performance Task* (CPT 3.0) [114] was used as a measure of inattentiveness, impulsivity, sustained attention, and vigilance. 

Declarative memory (administered at baseline, post-intervention, and at three months post-intervention for the GMT and WL conditions): The *California Verbal Learning Test II* (CVLT-II) [115] is a multiple-trial, word list learning task, which provides indices of immediate and delayed memory performance, interference learning, and recognition. 

#### 2.4.3. Subjective Cognition

The *Cognitive Failures Questionnaire* (CFQ) [116,117] is a 25-item self-report measure that captures daily errors in distractibility, blunders, names, and memory. The CFQ has been demonstrated to have good construct validity and internal consistency (α = 0.76–0.86) for its four subscales [117]. The CFQ was assessed at all clinician-administered assessment time points (i.e., baseline, post-intervention, and at three months post-intervention for the GMT and WL conditions) and during the GMT intervention (i.e., initial, third, sixth, and final GMT sessions) for those who initially were allocated to the GMT condition or who elected to participate in GMT following their participation in the WL condition.

#### 2.4.4. Functional Outcomes

The *World Health Organization Disability Assessment Schedule 2.0* (WHODAS 2.0) [118] was administered to assess individuals’ functioning across six domains, including cognition, mobility, self-care, getting along with others, life activities, and participation in the community. It has demonstrated high internal consistency across its six domains (α = 0.94–0.96), good test–retest reliability, and good convergence with other comparable measures [118]. It was administered during all clinician-administered assessment time points.

#### 2.4.5. Self-Report Symptom Measures

The *PTSD Checklist for DSM-5* (PCL-5) [102] was administered to participants to assess the severity of PTSD symptoms according to the diagnostic criteria outlined in the DSM-5 [1]. The symptom domains included intrusive symptoms, avoidance, negative alterations in mood and cognitions, and alterations in arousal and reactivity [1,102]. Participants rated the severity of their symptoms in the past month on a Likert scale from 0 (*not at all*) to 4 (*extremely*). A cut-point of 33 is suggested to indicate a probable PTSD diagnosis [119]. It has demonstrated high internal consistency among military (α = 0.95) [119] and veteran (α = 0.95) [120] samples. This measure was administered at all clinician-administered assessment time points and during the GMT intervention. 

The *Difficulties in Emotion Regulation Scale* (DERS) [10] is a 36-item self-report measure, which assesses difficulties with ER across six domains, including difficulties with accepting negative emotions; difficulties completing tasks or goals due to negative emotions; difficulties with controlling impulses while experiencing distressing emotions; difficulties with awareness of emotional experiences; negative beliefs regarding the ability to regulate emotions; and difficulties with insights regarding emotions. Higher scores indicated greater dysfunction with ER. The DERS was shown to have good psychometric properties, including internal consistency (α = 0.93) and construct validity [10]. This measure was administered at all clinician-administered assessment time points for the GMT and WL conditions. It also was administered during the GMT intervention. 

The *Multiscale Dissociation Inventory* (MDI) [121] is a 30-item self-report measure, which assesses six domains of dissociative symptoms over the past month, including disengagement, depersonalization, derealization, emotional constriction, memory disturbance, and identity dissociation. The MDI Total score has been demonstrated to have high internal consistency (α = 0.96) [121]. This measure also was administered at all clinician-administered assessment time points and during the GMT intervention.

The *Beck Depression Inventory-II* (BDI-II) [122] is a 21-item self-report questionnaire that assesses the presence and severity of symptoms of depression over the past 30 days. Symptoms assessed include hopelessness and irritability, feelings of guilt or feelings of being punished, as well as physical symptoms such as fatigue, weight loss, and loss of interest in sex. Respondents rate their symptoms on a Likert scale from 0 to 3, with higher scores reflecting greater symptom severity. The BDI-II has been demonstrated to have high internal consistency in psychiatric outpatients (α = 0.91) [123] and adequate convergent and discriminant validity [122]. The BDI-II was administered at all clinician-administered testing sessions and during the GMT intervention. 

The *Beck Anxiety Inventory* (BAI) [124] is a 21-item self-report questionnaire that assesses anxiety symptoms over the past 30 days. The items consist of common symptoms of anxiety, such as numbness, tingling, sweating, and fear of perceived catastrophic outcomes. Participants rate their symptoms on a four-point Likert Scale from 0 (*not at all)* to 3 (*severely*). It has been demonstrated to have excellent reliability in psychiatric outpatient samples (α = 0.92) [125]. It was administered at all clinician-administered testing sessions and during the GMT intervention. 

### 2.5. Statistical Methods

Data were analyzed using IBM SPSS Version 26.0. Independent samples *t*-tests and chi-square tests were used to analyze differences in demographic, clinical, neuropsychological, and self-report variables between WL and GMT groups at baseline. 

To assess our primary objectives, a series of 2 (time) × 2 (group) ANOVAs were conducted to assess the differences between the WL and GMT conditions from pre- to post-intervention, with analyses completed on participants who did not have any missing data from pre- to post-intervention for each measure of interest (i.e., modified intention-to-treat analysis). Subsequently, sample sizes are indicated for each measure and its analysis. Effect sizes were reported as partial eta-squared (interpreted as small = 0.01, medium = 0.10, and large = 0.25) for the neuropsychological, subjective cognition, functional, and self-report symptom measures. Given the large number of neuropsychological and self-report variables available from this comprehensive battery relative to the small number of participants in the pilot sample, the clinician-administered 3-month assessment time point was excluded from the primary analyses to minimize the risk of type I error. Moreover, due to the pilot nature of this study, the results of the ANOVAs were followed up with simple main effects analyses based on significant main effects (e.g., time or condition), observations of effect sizes, or inspection of means and SDs between the groups, as the pilot sample may not have been large enough in every case to assess results based on *p*-values alone.

To evaluate our secondary objectives in examining the trajectory of self-reported symptom changes and the effects of baseline variables on the trajectory of symptom changes during the GMT intervention, a series of one- and two-level hierarchical linear models was conducted. Hierarchical linear modelling (HLM) [126] was used given its ability to evaluate individual differences in the trajectory of change over time, and because it can accommodate missing data at level 1. The restricted maximum likelihood approach was used as the method for parameter estimation. The primary outcome variables were the CFQ, PCL-5, DERS, MDI, BDI-II, and BAI total scores, with the assessment time point as a level 1 predictor. Baseline PCL-5, DERS, MDI, BDI-II, and BAI total scores were used as predictors at level 2 in subsequent analyses, and level 2 variables were centred around the grand mean. All analyses assessed changes from baseline to post-intervention across six time points with the unit of time standardized to one week. Specifically, these analyses included all participants who participated in the GMT intervention (i.e., participants who were allocated to the GMT condition and those WL participants who agreed to participate in GMT following their wait period) and included their self-report assessments before, during, and after the GMT intervention. The one-level models assessed the trajectory of change in a single variable over time, whereas each of the two-level models assessed the effect of a level 2 predictor on the trajectory of change in the self-report symptom measures over time. Individuals missing greater than 30% of the data were excluded from the analysis. Analyses were completed using the HLM 7.0 statistical program. 

## 3. Results

The participants’ mean age across the WL and GMT conditions was 44.25 (*SD* = 7.50). Further, the mean years of education for the sample was 17.27 (*SD* = 2.87). Within the WL and GMT conditions, 77.8% and 90% of the participants, respectively, met the diagnostic criteria for PTSD according to the CAPS-5. Overall, 85.0% of the participants met the criteria for a diagnosis of PTSD across both samples. 

With respect to the pre-morbid IQ (i.e., WTAR Estimated IQ), participants in the WL and GMT conditions had mean IQ scores of 113.44 (*SD* = 6.08) and 113.32 (*SD* = 5.38), respectively. Further, the mean estimated IQs (i.e., WASI-II FSIQ) within the WL and GMT conditions were 105.56 (*SD* = 16.00) and 108.45 (*SD* = 14.13), respectively. Collectively, these pre-morbid and estimated IQ scores represent scores between the average and high average range. 

No significant differences emerged for the demographic and clinical characteristics between the WL and GMT conditions at baseline. All variables were assessed for homogeneity of variance (Levene’s test) and normality (using Kolmogorov–Smirnov and Shapiro-Wilk tests) prior to analyses being conducted. There were several violations of homogeneity of variance and normality (i.e., *p* < 0.05). Transformation of variables were completed [127]; however, the normality and homogeneity of variance were not improved. Therefore, the results of the non-transformed variables are reported. Subsequently, cautious interpretation of the findings is warranted.

Importantly, no adverse effects were reported as a result of participating in GMT.

### 3.1. Pre- and Post-Analysis of Neuropsychological Assessment Performance

Means and *SD*s for the pre-treatment neuropsychological assessment performance in each of the WL and GMT groups are presented in Table 2. A series of *t*-tests were performed on these data to ensure no baseline differences in cognitive performance between groups. All results were insignificant (*p* = 0.099 to 0.996). 

The results of the 2 × 2 ANOVAs for the neuropsychological assessments are reported in Table 3. 

#### 3.1.1. Tests of Executive Functioning, Processing Speed, and Attention

*COWAT*. There was a main effect of time for the FAS subtest (*F*(1, 27) = 9.044, *p =* 0.006, *η*^2^*_p_* = 0.251), indicating improved task performance for both the GMT and WL conditions from pre- to post-intervention. Simple main effects analyses revealed that while participants’ scores in the GMT condition improved significantly (*F*(1, 27) = 7.587, *p* = 0.010, *η*^2^*_p_* = 0.219) from pre- to post-intervention, participants’ scores in the WL condition did not (*F*(1, 27) = 2.768, *p* = 0.108, *η*^2^*_p_* = 0.093). This suggests that GMT had a medium effect on verbal fluency. There were no significant interactions or main effects for the COWAT Animals subtest (*p*s = 0.343 to 0.883).

*Stroop Color and Word Test*. The results of the 2 × 2 ANOVA for the Stroop Color and Word Test demonstrated significant main effects of time in the color subtest (*F*(1, 27) = 5.137, *p =* 0.032, *η*^2^*_p_* = 0.160), the color–word subtest (*F*(1, 27) = 12.477, *p =* 0.002, *η*^2^*_p_* = 0.316), and the interference score (*F*(1, 27) = 9.402, *p =* 0.005, *η*^2^*_p_* = 0.258), suggesting improvements for both groups from pre- to post-intervention. Simple main effects analyses were carried out. Notably, the results demonstrated that across the color (*F*(1, 27) = 4.982, *p =* 0.034, *η*^2^*_p_* = 0.156), color–word (*F*(1, 27) = 11.043, *p* = 0.003, *η*^2^*_p_* = 0.290), and interference (*F*(1, 27) = 6.582, *p* = 0.016, *η*^2^*_p_* = 0.196) scores, individuals’ scores within the WL condition improved significantly, while those in the GMT condition (*p*s = 0.417, 0.149, and 0.101, respectively) did not.

*DKEFS Tower Test*. There was a significant main effect of time (*F*(1, 26) = 11.186, *p =* 0.003, *η*^2^*_p_* = 0.301) and condition (*F*(1, 26) = 6.559, *p =* 0.017, *η*^2^*_p_* = 0.201) for the DKEFS first move time scaled score from pre- to post-intervention. Simple main effects analyses revealed that there was a significant increase in task initiation time from pre- to post-intervention for those participants in GMT (*F*(1, 26) = 15.722, *p* = 0.001, *η*^2^*_p_* = 0.377), but not for those in the WL condition (*F*(1, 26) = 1.479, *p =* 0.235, *η*^2^*_p_* = 0.054). This suggests that following GMT, individuals took longer to make their first move on the task than those in the WL condition. These findings represent a large effect. Moreover, there were also significant main effects of time for the time per move scaled score (*F*(1, 26) = 16.065, *p* < 0.001, *η*^2^*_p_* = 0.382) and rule violations (*F*(1, 26) = 5.935, *p =* 0.022, *η*^2^*_p_* = 0.186). Simple main effects analyses for the time per move scaled score demonstrated that there was a significant increase in the amount of time taken per move from pre-to post-intervention for those participants in GMT (*F*(1, 26) = 25.405, *p* < 0.001, *η*^2^*_p_* = 0.494), but not for those participants in the WL condition (*F*(1, 26) = 1.543, *p* = 0.225, *η*^2^*_p_* = 0.056). Moreover, the simple main effects for rule violations demonstrated that there was a significant decrease (i.e., less rule breaking) for the participants in the WL condition over time (*F*(1, 26) = 7.118, *p* = 0.013, *η*^2^*_p_* = 0.215), but not for GMT participants (*F*(1, 26) = 0.247, *p* = 0.623, *η*^2^*_p_* = 0.009). 

*WAIS-IV Digit Symbol Coding Subtest*. Analyses demonstrated that there was a significant main effect for time (*F*(1, 27) = 12.675, *p* = 0.001, *η*^2^*_p_* = 0.319), with simple main effects revealing significant increases in participants’ scores for both the GMT (*F*(1, 27) = 5.650, *p* = 0.025, *η*^2^*_p_* = 0.173) and WL (*F*(1, 27) = 7.079, *p* = 0.013, *η*^2^*_p_* = 0.208) conditions from pre- to post-intervention. 

*TMT*. There was a significant main effect of time (*F*(1, 25) = 7.581, *p* = 0.011, *η*^2^*_p_* = 0.233) for the TMT part B score from pre- to post-intervention. Notably, there was a simple main effect demonstrating a significant increase in scores for the participants in the GMT condition (*F*(1, 25) = 4.277, *p* = 0.049, *η*^2^*_p_* = 0.146), suggesting a reduction in the time taken to complete the task. Moreover, those in the WL condition also demonstrated an increase in their TMT part B scores over time; however, this result did not reach the alpha level of significance (*F*(1, 25) = 3.549, *p* = 0.071, *η*^2^*_p_* = 0.124). 

*CPT 3.0*. There were significant improvements (i.e., decreases) in commissions (*F*(1, 26) = 17.009, *p* < 0.001, *η*^2^*_p_* = 0.395) and detectability (*F*(1, 26) = 4.912, *p* = 0.036, *η*^2^*_p_* = 0.159) scores across time for both groups. Furthermore, there was a significant increase in perseverations across time for both groups (*F*(1, 26) = 6.259, *p* = 0.019, *η*^2^*_p_* = 0.194). With respect to commissions, simple main effects demonstrated significant decreases for both the GMT (*F*(1, 26) = 14.233, *p* = 0.001, *η*^2^*_p_* = 0.354) and WL (*F*(1, 26) = 5.437, *p* = 0.028, *η*^2^*_p_* = 0.173) conditions from pre- to post-intervention. Moreover, simple main effects analyses demonstrated a similar significant decrease for detectability from pre- to post-intervention within the GMT condition (*F*(1, 26) = 7.564, *p* = 0.011, *η*^2^*_p_* = 0.225), but not within the WL condition (*F*(1, 26) = 0.510, *p* = 0.481, *η*^2^*_p_* = 0.019). These findings represented a medium to large effect size. Finally, analyses of the simple main effect for perseverations demonstrated a significant increase in scores (i.e., a worsening) within the GMT condition (*F*(1, 26) = 6.360, *p* = 0.018, *η*^2^*_p_* = 0.197), but not the WL condition (*F*(1, 26) = 1.539, *p* = 0.226, *η*^2^*_p_* = 0.056). 

#### 3.1.2. Declarative Memory

*CVLT*. Results of the 2 × 2 ANOVAs for the CVLT also reveal several significant effects. Critically, there was a significant group x time interaction for the CVLT-II Short Delay Free Recall *Z* score (*F*(1, 27) = 7.963, *p* = 0.009, *η*^2^*_p_* = 0.228), with simple main effects indicating that participants in the GMT condition freely recalled a greater number of words from a list (*F*(1, 27) = 3.997, *p* = 0.056, *η*^2^*_p_*= 0.129). This finding, however, did not reach the alpha threshold for significance. Those in the WL condition experienced a decline in their ability to freely recall words from a list; however, this finding also did not reach the alpha threshold for significance (*F*(1, 27) = 4.076, *p* = 0.054, *η*^2^*_p_* = 0.131). Further, there was a significant main effect of time for the Trial 5 *Z* scores (*F*(1, 27) = 14.603, *p* = 0.001, *η*^2^*_p_* = 0.351). Here, simple main effects revealed a significant improvement for participants in the GMT condition (*F*(1, 27) = 14.264, *p* = 0.001, *η*^2^*_p_* = 0.346), with those participants in the WL condition also improving (*F*(1, 27) = 3.602, *p* = 0.068, *η*^2^*_p_* = 0.118), but did not reach the alpha level for significance. 

### 3.2. Pre and Post-Analysis of Subjective Cognition, Functioning, and Self-Report Symptom Measures

A series of *t*-tests were conducted to assess baseline differences between the GMT and WL conditions on each of the self-report variables. No significant differences were present between the two groups (*p*s = 0.057 to 0.292). The means and *SD*s for those participants who completed both pre- and post-intervention assessments on each self-report measure are reported in Table 4.

The results of the 2 × 2 ANOVAs for the subjective cognition, functional, and self-report symptom measures are reported in Table 5. Inspection of the means for the CFQ total score indicated a potential effect of the GMT intervention (i.e., an approximate eight-point decrease versus a two-point decrease in mean CFQ total scores between GMT and the WL condition; Table 4). Indeed, simple main effect analyses demonstrated that the CFQ total score for those participants in the GMT condition decreased significantly over time (*F*(1, 27) = 5.344, *p* = 0.029, *η*^2^*_p_* = 0.165), while it did not for those in the WL condition (*F*(1, 27) = 0.152, *p* = 0.699, *η*^2^*_p_* = 0.006), suggesting a medium effect of the GMT intervention on subjective cognitive function.

Moreover, the WHODAS score showed a significant group x time interaction, with simple main effects indicating that scores for the GMT condition significantly decreased over time (*F*(1, 26) = 11.39, *p* = 0.002, *η*^2^*_p_* = 0.305), while the WL group did not (*F*(1, 26) = 0.095, *p* = 0.760, *η*^2^*_p_* = 0.004). These results indicate a large positive effect of GMT on functioning.

The interaction for the PCL-5 total score approached significance (*F*(1, 23) = 3.641, *p* = 0.069, *η*^2^*_p_* = 0.137). Simple main effects showed that the PCL-5 total score for participants in the GMT condition decreased significantly over time (*F*(1, 23) = 5.63, *p* = 0.026, *η*^2^*_p_* = 0.197), while it did not for those in the WL condition (*F*(1, 23) = 0.199, *p* = 0.660, *η*^2^*_p_* = 0.009). This suggests a reduction in PTSD symptom severity following participation in the GMT intervention.

There also was a significant main effect of time for the DERS total score (*F*(1, 26) = 4.473, *p* = 0.044, *η*^2^*_p_* = 0.147; Table 5), suggesting that both groups experienced decreases in their DERS total scores from pre- to post-intervention. The results of the simple main effects revealed that participants in the GMT condition showed a significant reduction in DERS total score (*F*(1, 26) = 6.85, *p* = 0.015, *η*^2^*_p_* = 0.209); however, this finding was not observed for those participants in the WL condition (*F*(1, 26) = 0.371, *p* = 0.548, *η*^2^*_p_* = 0.014). These results indicate a medium effect of the GMT intervention on reductions in difficulties with ER. 

With respect to MDI total score, the interaction between time and intervention approached significance (*F*(1, 27) = 3.164, *p* = 0.087, *η*^2^*_p_* = 0.105). Here, simple main effects demonstrated that following their participation in the GMT condition, participants experienced significant reductions in MDI total scores (*F*(1, 27) = 4.223, *p* = 0.050, *η*^2^*_p_* = 0.135), whereas those participants in the WL condition did not (*F*(1, 27) = 0.424, *p* = 0.520, *η*^2^*_p_* = 0.015). This represented a moderate effect of GMT on dissociative symptoms. 

Finally, there also were significant main effects of time for BDI-II (*F*(1, 26) = 9.797, *p* = 0.004, *η*^2^*_p_* = 0.274) and BAI (*F*(1, 25) = 5.088, *p* = 0.033, *η*^2^*_p_* = 0.169) total scores across both conditions. Specifically, for the BDI-II total score, simple main effects revealed that those participants in the GMT condition showed a significant reduction in total score from pre- to post-intervention (*F*(1, 26) = 11.314, *p* = 0.002, *η*^2^*_p_* = 0.303), which was not found for those participants in the WL condition (*F*(1, 26) = 1.951, *p* = 0.174, *η*^2^*_p_* = 0.070). 

A similar result was observed for the BAI with reductions in BAI total scores for those participants in the GMT condition (*F*(1, 25) = 7.788, *p* = 0.010, *η*^2^*_p_* = 0.238), but not for those participants in the WL condition (*F*(1, 25) = 0.623, *p* = 0.437, *η*^2^*_p_* = 0.024). These results indicate medium to large effects for self-reported depressive and anxiety symptoms for those participants in the GMT condition. 

### 3.3. Trajectory of Change for Subjective Cognition and Self-Report Symptom Measures

Table 6 demonstrates the level 1 HLM analyses for the trajectory of subjective cognition and symptom severity changes over time for those individuals who participated in GMT (i.e., either initially randomized to GMT or those individuals from the WL who participated after their waiting period). With respect to subjective cognition, CFQ total scores significantly decreased across time (*b* = −1.44, *t*(33) = −2.72, *p* = 0.010). Furthermore, the PCL-5 total score (*b* = −2.23, *t*(33) = −5.97, *p* < 0.001), DERS total score (*b* = −2.36, *t*(33) = −3.89, *p* < 0.001), MDI total score (*b* = −1.24, *t*(33) = −3.70, *p* < 0.001), BDI-II total score (*b* = −1.39, *t*(33) = −4.56, *p* < 0.001), and BAI total score (*b* = −1.33, *t*(33) = −4.25, *p* < 0.001) also significantly decreased over time.

The findings for the level 2 HLMs assessing the effects of baseline variables on the trajectory of change in subjective cognition and self-reported symptoms over time are presented in Table 7. Here, the DERS total score significantly influenced the trajectory of the MDI total score over the duration of GMT, such that those with higher baseline DERS total scores experienced significantly greater reductions in MDI total scores over time (*b* = −0.02, *t*(30) = −2.11, *p* = 0.044). Similarly, the baseline BAI total score significantly influenced the trajectory of the CFQ total score over time (*b* = −0.08, *t*(31) = −2.14, *p* = 0.041). Although it did not reach the alpha level of significance of *p* = 0.05, an additional finding suggests that the MDI total score may influence the trajectory of the CFQ total score over time (*b* = −0.06, *t*(31) = −1.77, *p* = 0.087). Additional investigations of this trend within a more robust sample size may be warranted in future studies. No other investigated level 2 HLMs demonstrated significant findings. 

## 4. Discussion

The primary objective of this pilot RCT was to determine the effectiveness of GMT as compared to a WL condition in improving objective and subjective cognition, functioning, symptoms of PTSD, and symptoms associated with PTSD (i.e., difficulties with ER, dissociation, depression, and anxiety) in a sample of military personnel, veterans, and PSP. It was hypothesized that those participants randomized to the GMT condition would experience significant improvements in these outcomes relative to the WL condition. Generally, the results of this pilot RCT support these hypotheses. Specifically, relative to participants in the WL condition, participants in the GMT condition experienced significant improvements in areas of executive functioning, such as improvements in verbal fluency, planning, impulsivity, cognitive shifting, and discrimination of targets. Collectively, the effect sizes for these significant simple main effects ranged from medium to large, suggesting that the effects of the GMT intervention may be replicated within a larger-scale RCT. Moreover, the findings suggest that GMT may improve short-term declarative memory, as individuals in the GMT condition demonstrated a trending improvement in their ability to recall words from a list in comparison to those individuals in the WL condition. Future replications of this study in a larger sample size may further elucidate this finding. 

Notably, the significant improvements in objective cognition following the GMT intervention support previous research. For example, following completion of GMT, patients diagnosed with obsessive compulsive disorder demonstrated significant improvements in measures of problem-solving, planning, impulsivity, attention, and processing speed in comparison to patients who were randomized to a WL condition [98]. In another study, Boyd et al. (2019) demonstrated improvements on measures of executive functioning, planning, attention, and short-term declarative memory following a modified GMT protocol for individuals receiving concurrent inpatient trauma treatment [99]. Similarly, the current study found similar significant effects following the GMT intervention relative to the WL condition in tasks of executive functioning (e.g., verbal fluency, impulsivity, cognitive shifting, and discrimination of targets) and planning, as well as a trending improvement in short-term declarative memory. Critically, the findings of the current pilot RCT also demonstrate focused improvements in cognitive difficulties typically associated with PTSD, including executive functioning [26,29,30,31,32] and verbal learning, suggesting that GMT may be an effective intervention to help address these concerns among military personnel, veterans, and PSP with PTSD symptoms and symptoms associated with PTSD.

Although participants in the GMT condition did not experience significant improvements in their Stroop color, color–word, and interference scores relative to the WL condition, these specific findings also help to demonstrate support that the GMT intervention may improve executive functioning. GMT is designed to assist individuals with noticing attentional lapses and approaching tasks in a mindful manner, with the goal of reinstating executive cognitive control when there is a discrepancy between the individual’s goal and behaviour [59,89,128]. Given that the Stroop Color and Word Test instructs participants to complete the tasks as quickly as possible within a time limit [110,111], the lack of improvements in these scores for those participants in the GMT condition suggests individuals may have been more mindful while completing the task. Specifically, individuals in the GMT condition may have been checking that their goal of completing the task accurately matched with their behaviour, thereby slowing their response time, which subsequently did not lead to score improvements. Relative to the individuals in the GMT condition, the individuals in the WL condition did not receive such training and may have increased their speed on this task given their familiarity with it during the post-intervention testing. 

This purposeful slowing of response time while completing tasks may also explain the significant increase in scores (i.e., worsening) for CPT 3.0 perseverations for those individuals in the GMT condition. Perseverations may be attributed to slowed responses to preceding stimuli, as well as random responses (e.g., errors), anticipatory responses (e.g., guesses), or repeated responses, suggesting impulsivity [114]. As impulsivity improved following GMT (measured by the outcomes on the DKEFS Tower Test), the results of the CPT 3.0 perseverations score suggests that participants in the GMT condition may have slowed their responding. Subsequently, this may have caused individuals to miss the original target and respond too quickly to the next target in the task. Alternatively, there may be several other possibilities that may explain these findings, including anticipatory responses, repeated responses, and that these cognitive functions may not demonstrate significant change over the duration of the study trial. Subsequently, future research should continue to explore these findings for additional clarification.

Improvements in the other neuropsychological measures may be attributable to practice effects. For example, although individuals in the GMT condition improved significantly on TMT part B, the findings for those in the WL condition also demonstrated a trend towards score improvement. A similar trend was also observed on the WAIS-IV Digit Symbol Coding Subtest, CPT 3.0 commissions, and CVLT Trial 5 *Z* scores. Practice effects may also account for the improvements found on the DKEFS Tower Test rule violations measure for those in the WL condition, which was not found among those participants in the GMT condition. While there were no significant differences between the groups at baseline for this measure, the maximum score for rule violations in the WL condition was higher at baseline than the maximum score in the GMT condition (i.e., four errors versus two errors, respectfully). At the subsequent follow-up testing appointment, those individuals in the WL condition improved as they had a maximum score of two for rule violations, whereas those participants in the GMT condition continued to have a maximum score of two for rule violations. Consequently, this improvement in the WL condition’s rule violations may be attributable to practice effects, as there was no intervention administered to this group and their improvement in this measure may be attributable to their previous experience and familiarity with the DKEFS Tower Test. 

With respect to subjective cognition and self-reported functioning, participants in the GMT condition also experienced significant improvements from pre- to post-testing in both domains, whereas participants in the WL condition did not. Generally, PTSD is associated with poorer social and occupational functioning [129,130], as well as reduced quality of life [131]. More specifically, previous research has linked poorer subjective cognitive functioning to higher levels of psychological distress [36,38,39], poorer functioning [38], and poorer quality of life [36,39,132] among individuals with PTSD. Previous research also has found that objective measures of cognition are negatively associated with physical [40], social, and occupational functioning [40,41] among military personnel and veterans with PTSD. Considering this previous research, the current study’s findings demonstrate that not only may GMT prove to be beneficial in assisting individuals’ objective and subjective cognitive functioning, but also that these improvements in cognition may translate to improvements in functioning for military personnel, veterans, and PSP. Future work should aim to examine the specific domains of functioning (e.g., social, occupational, daily, etc.) affected following participation in GMT, as well as whether quality of life also shows a similar improvement following the GMT intervention. Additional studies should also aim to examine whether GMT may be a useful intervention in assisting military personnel, veterans, and PSP during return-to-work following a medical leave of absence due to PTSD. Notably, this represents a significantly understudied area of clinical research as the factors that contribute to a successful return to work for military personnel, veterans, and PSP have remained largely unknown.

Improvements in psychological symptoms following participation in the GMT intervention relative to the WL condition were also found. Specifically, simple main effects demonstrated that participants experienced improvements in PTSD symptom severity, difficulties with ER, dissociative symptom severity, depressive symptoms, and anxiety symptoms in the GMT condition, but not in the WL condition. These findings also demonstrated a medium to large effect size, suggesting again that these findings may be replicated within a larger-scale clinical trial. These results may also suggest that along with improvements in objective and subjective cognitive difficulties, improvements in PTSD symptoms and symptoms associated with PTSD may be indirectly targeted through a cognitive remediation intervention. This implies that GMT may be a useful adjunctive treatment for individuals experiencing PTSD and its related psychological symptoms. Importantly, these results support the use of a top-down cognitive remediation approach to address objective and subjective cognitive difficulties, as well as PTSD and PTSD-related psychological symptoms. Top-down cognitive remediation targets higher-order neurocognitive abilities, such as executive functioning, which can assist in the improvement of other cognitive functions and in the generalization of these improvements to various contexts [59,89,133,134]. By employing such an approach, the aim is to not only improve specific cognitive functions but to also ameliorate any downstream dysfunction associated with the neural regions and neural circuitry responsible for these cognitive functions. Specifically, indirect improvements in tasks of daily functioning and other psychological symptoms following the implementation of a top-down cognitive remediation intervention were expected given that previous studies have demonstrated similar findings [97,135]. Moreover, our results support previous meta-analytic findings, which suggest GMT is associated with small to medium improvements in objective and subjective measures of executive functioning, as well as improvements in tasks of daily living and other mental health symptoms [97]. 

A secondary objective of the study was to examine the trajectory of self-reported symptom change over the course of the GMT intervention for those individuals who were initially randomized to the GMT condition, as well as those individuals in the WL condition who elected to participate in GMT following their waiting period. Here, it was hypothesized that the trajectory of symptoms would significantly decrease over time. Following level 1 HLM analyses, the results indicated that there were significant declines in self-reported cognitive difficulties, as well as PTSD symptom severity, difficulties with ER, dissociative symptom severity, depression symptom severity, and anxiety symptom severity. Notably, these findings further support the assertion that GMT may be a useful adjunctive treatment for PTSD, as well as for other psychological symptoms, given the significant decline in the severity of these symptoms across time. Future studies are needed to elucidate whether such symptom declines are comparable to those gained with current evidence-based treatments, such as cognitive processing therapy and prolonged exposure therapy.

Additional exploratory analyses were conducted to examine the effects of baseline self-reported symptoms (e.g., subjective cognitive difficulties, PTSD symptoms, and other psychological symptoms) on the trajectory of specific symptoms over time. Here, the results demonstrated that difficulties with ER significantly influenced the trajectory of dissociative symptoms across the GMT intervention, such that those participants with greater baseline difficulties with ER experienced significantly greater reductions in dissociative symptoms over time. Dissociative symptoms have been proposed as an ER strategy during and following traumatic events [15], as they allow individuals to detach from intense and distressing emotions. Subsequently, these findings lend support to this established relationship, as improvements in ER significantly influenced the trajectory of dissociative symptoms over time. Moreover, this also supports the notion that individuals with more severe difficulties with ER may still experience benefits from participating in GMT and that these symptoms do not appear to interfere with other symptom improvements. 

The analyses also explored whether baseline dissociative symptoms influenced the trajectory of self-reported cognitive difficulties over the course of the GMT intervention. Although these results did not reach the significance threshold (i.e., *p* = 0.05), they trended towards significance. Notably, previous research suggests a strong relation between dissociative symptoms and cognitive functioning [33,34,35,136], such that increased dissociative symptom severity is associated with heightened difficulties with verbal memory [34], visual memory [34,35], attention [35], executive functioning, and autobiographical memory. Further studies involving larger sample sizes are required to confirm the specificity of these effects.

Additional analyses examined whether baseline anxiety symptom severity influenced the trajectory of subjective cognitive difficulties over the course of the GMT intervention. Notably, these results were significant, such that those participants who began the GMT intervention with higher anxiety scores showed greater reductions in subjective cognitive difficulties over the duration of GMT. Numerous studies document the relation between anxiety and cognitive difficulties, where anxiety symptoms, such as worry, can impair executive functions such as problem-solving [137] and inhibition [138,139], as well as impair attention [140] and working memory [138]. Clinician-rated severity of difficulties with concentration associated with anxiety symptoms appears further to mediate the relation between subjective reports of worry and clinician-rated severity of anxiety symptoms [141]. Here, it is thought that anxiety increases cognitive demand, thereby interfering with other cognitive functions, including executive functioning and attention [142,143,144]. In line with previous work, the current findings suggest that improvements in reported anxiety symptoms reduce subjective cognitive difficulties over time. 

Exploratory analyses also examined the effects of baseline symptoms on the trajectory of change in subjective cognition, as well as the severity of other psychological symptoms. Specifically, we examined whether difficulties with ER influenced the trajectory of self-reported cognitive difficulties, as well as PTSD, depression, and anxiety symptom severity. We also explored whether PTSD symptom severity and depression symptom severity influenced the trajectory of self-reported cognitive difficulties. No such relations emerged. These findings were somewhat unexpected given the reported associations between difficulties with ER and cognitive dysfunction [15,50,51,60,61,62], difficulties with ER and psychological symptoms [10,16,17,145], PTSD symptom severity and self-reported cognitive difficulties [28,32], and depression and self-reported cognitive difficulties [146]. Additional research with a larger sample is clearly warranted. This work is ongoing in our laboratory.

The results of the study must be interpreted with caution. As this is a pilot study, it involved a relatively small sample size. The large number of analyses conducted may have increased the probability of type I error leading to erroneous conclusions. To mitigate this potential error, the analyses chosen were based on previous findings within the literature and specific hypothesis were explored. Analyses also were limited to pre- and post-intervention to ensure the retention of the largest possible sample size. An additional limitation of the study is that the ANOVA analyses were followed up with simple main effects, not only when there were significant interactions, but also if there were significant main effects or inspection of the effect sizes and means and SDs between the GMT and WL conditions suggested further exploration. Accordingly, despite very promising signals, the results of the study should be interpreted with caution. Analyses also were conducted on data in which there were several violations of homogeneity of variance and normality. Transformation of these data did not improve the normality or homogeneity of variance, and parametric analyses were conducted on the non-transformed variables. 

With respect to the study design, the repeated use of certain neuropsychological assessment measures (e.g., DKEFS Tower Test, WAIS-IV Digit Symbol Coding Subtest, TMT, etc.) may have positively influenced the test outcome due to practice. Where appropriate, outcomes which may be attributable to practice effects were indicated. Critically, these practice effects would be expected to be equivalent across the active treatment and wait-list groups. An additional limitation of the study design is that individuals could have been administered the same self-report measures as those in the GMT condition during the WL condition’s nine-week waiting period. Doing so would have allowed for comparisons between the WL and the GMT conditions using HLM analyses. Future work should consider implementing this methodology. 

A strength of this study, however, was its status as an effectiveness pilot RCT designed to be inclusive of participants with a spectrum of PTSD and co-morbid psychological symptoms to mimic the diversity of symptom presentation found within clinical settings. This factor coupled with the strong study findings suggest that improvements associated with GMT may generalize to military personnel, veterans, and PSP seen under real-world conditions with varying symptom severity of PTSD and other psychological comorbidities and symptoms. Future work should aim to replicate these findings within a larger sample size to determine whether these preliminary findings regarding the effectiveness of GMT continue to hold. A larger sample size would allow analyses to determine the durability of these findings. Future research may also compare GMT to an active WL condition (e.g., psychoeducation group discussing brain and cognitive changes associated with PTSD; this work is underway in our laboratory). This design would assist in determining whether GMT or the process of being in a treatment group with clinicians and other group members with similar symptoms contributes to the improvements found following participation in the GMT intervention. Finally, given that GMT targets objective cognitive difficulties and that PTSD is associated with alterations in cognitive functioning that may stem from altered neural functioning and circuitry, it would be helpful to conduct a RCT that includes neuroimaging pre- and post-treatment and in comparison, to a matched control condition. This design would allow us to assess any functional or structural brain changes associated with participation in GMT. 

## 5. Conclusions

On balance, this pilot RCT is the first to examine whether GMT is an effective cognitive remediation intervention among military personnel, veterans, and PSP with symptoms of PTSD and co-symptoms commonly associated with this condition. The findings of the study suggest that not only do objective and subjective measures of cognition improve following GMT, but also that functioning and symptoms of PTSD, difficulties with ER, dissociation, depression, and anxiety also show improvement. Given that cognitive difficulties persist for approximately 25% of patients following treatment of PTSD [46], as well as the devastating functional impacts associated with PTSD and cognitive dysfunction [40,41], these findings are promising, suggesting the potential utility of GMT as an adjunctive treatment for this condition.

## Figures and Tables

**Table 1:** Demographic and clinical characteristics of eligible participants.

Characteristics	WL (*n* = 18)	GMT (*n* = 22)
Demographic characteristics		
Sex (female: male)	3:15	10:12
Age (Mean, (SD))	44.61 (8.54)	43.95 (6.73)
Race (Caucasian/Aboriginal/Hispanic)	15:2:1	22:1:0
Marital Status	% of Sample	
Single	5.6	13.6
Married or common law	72.2	77.3
Separated/Divorced	16.7	0
Long-term relationship	5.6	9.1
Highest Level of Education Completed		
Completed high school	5.6	0
Some college or university	5.6	9.1
Completed college or university	77.8	77.3
Some graduate level education	5.6	4.5
Completed graduate degree	5.6	9.1
Current Employment Status		
Working full time	44.4	27.3
Working part time	5.6	0
Medical Leave/Long-term Disability	22.2	22.7
Modified duties/Return to work	0	4.6
On leave/WSIB	22.2	27.3
Not currently employed	5.6	9.1
Other	0	9.1
Military, Veteran, or Public Safety Personnel Status		
Military	0	18.2
Veteran	5.6	13.6
Public Safety Personnel	94.4	95.5
Both	0	27.3
Clinical characteristics		
CAPS-5—Severity Score (Mean, (SD))	36.00 (14.33)	40.86 (12.67)
CAPS-5—PTSD Criteria Met (% of Sample)	77.8	90
Additional M.I.N.I. 7.0.2 diagnoses	% of Sample	
Major Depressive Disorder	61.1	63.6
Generalized Anxiety Disorder	27.8	54.5
Social Anxiety Disorder	27.8	50.0
Panic Disorder	16.7	27.3
Agoraphobia	11.1	40.9
Alcohol Use Disorder, Past 12 Months	5.6	13.64
Substance Use Disorder, Past 12 Months	0	4.55
Binge Eating Disorder	15.0	4.55
IQ	Mean (SD)	
WTAR Estimated IQ	113.44 (6.08)	113.32 (5.38)
WASI-II FSIQ	105.56 (16.00)	108.45 (14.13)

*Note.* WL = Waitlist; GMT = Goal Management Training; WSIB = Workplace Safety and Insurance Board; CAPS-5 = Clinician-Administered PTSD Scale for DSM-5; IQ = Intelligence Quotient; WTAR = Wechsler Test of Adult Reading; WASI-II = Wechsler Abbreviated Scale of Intelligence, Second Edition; M.I.N.I. 7.0.2 = Mini International Neuropsychiatric Interview 7.0.2.

**Table 2:** Means and SDs for baseline neuropsychological assessment performance between WL and GMT.

Variable	Group	*n*	Mean	*SD*
COWAT				
FAS T Score	WL	18	42.28	8.98
	GMT	22	41.41	10.25
Animals T Score	WL	18	48.28	12.84
	GMT	22	49.32	12.81
Stroop Color and Word Test				
Word T-Score	WL	18	36.78	10.64
	GMT	22	37.55	11.28
Color T-Score	WL	18	39.17	10.43
	GMT	22	41.14	10.74
Color-Word Trial T Score	WL	18	43.44	8.00
	GMT	22	44.09	11.17
Interference T-Score	WL	18	49.56	6.78
	GMT	22	49.41	6.96
DKEFS Tower Test				
Total Scaled Score	WL	18	11.22	2.53
	GMT	22	11.45	2.18
First Move Time Scaled Score	WL	18	10.61	2.57
	GMT	22	9.23	2.58
Time Per Move Scaled Score	WL	18	9.61	2.85
	GMT	22	8.82	3.51
Move Accuracy Scaled Score	WL	18	9.17	2.68
	GMT	22	9.50	3.40
Rule Violations	WL	18	0.78	1.16
	GMT	22	0.45	0.67
WAIS-IV				
Digit Symbol Coding Scaled Score	WL	18	9.78	3.00
	GMT	22	9.36	2.26
TMT				
TMT Part A T-Score	WL	17	50.00	9.25
	GMT	21	47.43	11.47
TMT Part B T-Score	WL	16	46.88	9.16
	GMT	21	44.67	10.73
CPT 3.0 ^a^				
Omissions T Score	WL	18	47.94	8.35
	GMT	22	47.95	5.10
Commissions T Score	WL	18	51.89	7.75
	GMT	22	52.27	8.95
Detectability T Score	WL	18	48.94	6.03
	GMT	22	50.27	8.40
Hit Reaction Time T Score	WL	18	46.83	8.58
	GMT	22	43.68	7.93
Variability T Score	WL	18	50.50	11.13
	GMT	22	52.00	7.80
Perseverations T Score	WL	18	45.94	3.12
	GMT	22	48.55	7.58
CVLT-II				
Trial 1 *Z* Score	WL	18	−0.22	1.73
	GMT	22	0.52	1.15
Trials 5 *Z* Score	WL	18	−0.14	0.78
	GMT	22	−0.21	0.97
Trial 1-15 *Z* Score	WL	18	50.61	10.88
	GMT	22	55.68	10.37
Trial B *Z* Score	WL	18	0.14	1.61
	GMT	22	0.02	0.88
Short-Delay Free Recall *Z* Score	WL	18	0.17	0.73
	GMT	22	−0.14	1.20
Short-Delay Cued Recall *Z* Score	WL	18	0.17	0.82
	GMT	22	0.09	1.07
Long-Delay Free Recall *Z* Score	WL	18	0.11	0.96
	GMT	22	−0.07	1.15
Long-Delay Cued Recall *Z* Score	WL	18	−0.08	0.77
	GMT	22	−0.09	0.93
Repetitions *Z* Score	WL	18	0.14	1.30
	GMT	22	0.07	1.13
Intrusions *Z* Score	WL	18	0.23	1.53
	GMT	22	−0.16	1.00
Discriminability *Z* Score	WL	18	−0.06	1.14
	GMT	22	0.25	1.09

*Note.* WL = Waitlist; GMT = Goal Management Training; COWAT = Controlled Oral Word Association Task; FAS represents the letters “F”, “A”, and “S” for the COWAT subtest; DKEFS = Delis-Kaplan Executive Function System; WAIS-IV = Wechsler Adult Intelligence Scale Fourth Edition; TMT = Trail Making Test; CPT 3.0 = Conner’s Continuous Performance Test—Third Edition; CVLT-II = California Verbal Learning Test—Second Edition. ^a^ Higher T scores for CPT 3.0 indicates poorer performance.

**Table 3:** 2 × 2 ANOVA table for neuropsychological assessments.

Tests of Executive Functioning, Processing Speed, and Attention					
Neuropsychological Test	Neuropsychological Subtest	Source	*F*	*p*	*η* ^2^ *_p_*
COWAT ^a^	FAS T Score	Time	9.044	0.006 **	0.251
		Condition	0.373	0.547	0.014
		Time * Condition	0.149	0.703	0.005
	Animals T Score	Time	0.932	0.343	0.033
		Condition	0.664	0.422	0.024
		Time * Condition	0.022	0.883	0.001
Stroop Color and Word Test ^a^	Word T Score	Time	3.352	0.078	0.110
		Condition	0.002	0.968	0.000
		Time * Condition	0.056	0.815	0.002
	Color T Score	Time	5.137	0.032 *	0.160
		Condition	0.101	0.754	0.004
		Time * Condition	1.564	0.222	0.055
	Color-Word T Score	Time	12.477	0.002 **	0.316
		Condition	0.016	0.901	0.001
		Time * Condition	2.903	0.100	0.097
	Interference T Score	Time	9.402	0.005 **	0.258
		Condition	0.076	0.785	0.003
		Time * Condition	0.953	0.338	0.034
DKEFS Tower Test ^b^	Total Score Scaled Score	Time	3.557	0.071	0.120
		Condition	0.251	0.621	0.010
		Time * Condition	0.002	0.964	0.000
	First Move Time Scaled Score	Time	11.186	0.003 **	0.301
		Condition	6.559	0.017 *	0.201
		Time * Condition	1.945	0.175	0.070
	Time Per Move Scaled Score	Time	16.065	0.000 **	0.382
		Condition	1.550	0.224	0.056
		Time * Condition	4.066	0.054	0.135
	Move Accuracy Scaled Score	Time	0.035	0.854	0.001
		Condition	0.812	0.376	0.030
		Time * Condition	0.730	0.401	0.027
	Rule Violations	Time	5.935	0.022 *	0.186
		Condition	1.335	0.258	0.049
		Time * Condition	3.393	0.077	0.115
WAIS-IV ^a^	Digit Symbol Coding Scaled Score	Time	12.675	0.001 **	0.319
		Condition	1.054	0.314	0.038
		Time * Condition	0.400	0.533	0.015
TMT ^c^	TMT Part A T Score	Time	0.604	0.445	0.024
		Condition	0.223	0.641	0.009
		Time * Condition	0.198	0.660	0.008
	TMT Part B T Score	Time	7.581	0.011 *	0.233
		Condition	0.025	0.875	0.001
		Time * Condition	0.056	0.815	0.002
CPT 3.0 ^b^	Omissions T Score	Time	0.018	0.895	0.001
		Condition	0.005	0.946	0.000
		Time * Condition	2.133	0.156	0.076
	Commissions T Score	Time	17.009	0.000 **	0.395
		Condition	0.012	0.915	0.000
		Time * Condition	0.148	0.703	0.006
	Detectability T Score	Time	4.912	0.036*	0.159
		Condition	0.328	0.572	0.012
		Time * Condition	1.147	0.294	0.042
	Hit Reaction Time T Score	Time	0.235	0.632	0.009
		Condition	0.107	0.746	0.004
		Time * Condition	2.078	0.161	0.074
	Variability T Score	Time	0.450	0.508	0.017
		Condition	1.086	0.307	0.040
		Time * Condition	0.375	0.546	0.014
	Perseverations T Score	Time	6.259	0.019 *	0.194
		Condition	0.916	0.347	0.034
		Time * Condition	0.263	0.613	0.010
Tests of Declarative Memory					
CVLT-II ^a^	Trial 1 *Z* Score	Time	0.032	0.860	0.001
		Condition	2.997	0.095	0.100
		Time * Condition	0.228	0.637	0.008
	Trial 5 *Z* Score	Time	14.603	0.001 **	0.351
		Condition	0.293	0.593	0.011
		Time * Condition	0.690	0.413	0.025
	Trial 1-5 T Score	Time	0.592	0.448	0.021
		Condition	1.668	0.207	0.058
		Time * Condition	1.247	0.274	0.044
	Trial B *Z* Score	Time	0.944	0.340	0.034
		Condition	0.494	0.488	0.018
		Time * Condition	2.976	0.096	0.099
	Short Delay Free Recall *Z* Score	Time	0.129	0.722	0.005
		Condition	0.356	0.556	0.013
		Time * Condition	7.963	0.009 **	0.228
	Short Delay Cued Recall *Z* Score	Time	0.217	0.645	0.008
		Condition	0.880	0.357	0.032
		Time * Condition	3.530	0.071	0.116
	Long Delay Free Recall *Z* Score	Time	0.475	0.496	0.017
		Condition	0.426	0.519	0.016
		Time * Condition	1.955	0.173	0.068
	Long Delay Cued Recall *Z* Score	Time	0.824	0.372	0.030
		Condition	1.621	0.214	0.057
		Time * Condition	2.372	0.135	0.081
	Repetitions *Z* Score	Time	0.107	0.746	0.004
		Condition	0.884	0.355	0.032
		Time * Condition	0.604	0.444	0.022
	Intrusions *Z* Score	Time	2.495	0.126	0.085
		Condition	0.480	0.494	0.017
		Time * Condition	0.782	0.384	0.028
	Discriminability *Z* Score	Time	1.838	0.186	0.064
		Condition	1.161	0.291	0.041
		Time * Condition	0.472	0.498	0.017

*Note.* GMT = Goal Management Training; WL = Waitlist; COWAT = Controlled Oral Word Association Task; FAS represents the letters “F”, “A”, and “S” for the COWAT subtest; DKEFS = Delis-Kaplan Executive Function System; WAIS-IV = Wechsler Adult Intelligence Scale Fourth Edition; TMT = Trail Making Test; CPT 3.0 = Conner’s Continuous Performance Test – Third Edition; CVLT-II = California Verbal Learning Test – Second Edition. ^a^ GMT *n* = 18, WL *n* = 11. ^b^ GMT *n* = 18, WL *n* = 10. ^c^ GMT *n* = 17, WL *n* = 10. Note: * *p* < 0.05. ** *p* < 0.01.

**Table 4:** Means and SDs for pre- and post-intervention scores for the subjective cognition, functioning, and self-report symptom measures for WL and GMT groups.

Measures	Group (*n*) ^a^	Pre-Intervention		Post-Intervention	
		Mean	*SD*	Mean	*SD*
CFQ	WL (11)	50.82	12.55	49.09	12.79
	GMT (18)	54.50	20.54	46.50	15.74
WHODAS Score	WL (11)	31.44	11.35	32.39	14.51
	GMT (17)	42.03	19.67	33.70	13.79
PCL-5 Total Score	WL (11)	38.55	15.92	39.82	15.77
	GMT (14)	38.71	12.08	32.71	14.90
DERS Total Score	WL (11)	94.09	23.79	90.91	24.00
	GMT (17)	101.53	23.87	90.53	23.55
MDI Total Score	WL (11)	50.36	15.48	52.82	16.67
	GMT (18)	55.61	16.68	49.56	12.52
BDI-II Total Score	WL (10)	25.30	8.31	21.40	6.62
	GMT (18)	28.11	12.10	21.11	11.21
BAI Total Score	WL (9)	26.33	14.20	23.56	12.08
	GMT (18)	25.44	12.55	18.50	10.15

*Note.* WL = Waitlist; GMT = Goal Management Training; CFQ = Cognitive Failures Questionnaire; WHODAS = World Health Organization Disability Assessment Schedule 2.0; PCL-5 = PTSD Checklist for DSM-5; DERS = Difficulties in Emotion Regulation Scale; MDI = Multiscale Dissociation Inventory; BDI-II = Beck Depression Inventory II; BAI = Beck Anxiety Inventory. ^a^ Sample sizes reflect the total number of participants who did not have any missing data for the indicated measure at pre- and post-intervention.

**Table 5:** The 2 × 2 ANOVA tables for the subjective cognition, functioning, and self-report symptom measures.

Measures	Source	*F*	*p*	*η* ^2^ *_p_*
CFQ Total Score	Time	2.997	0.095	0.100
	Condition	0.009	0.924	0.000
	Time * Condition	1.246	0.274	0.044
WHODAS Score	Time	3.516	0.072	0.119
	Condition	1.092	0.306	0.040
	Time * Condition	5.552	0.026	0.176
PCL-5 Total Score	Time	1.538	0.227	0.063
	Condition	0.388	0.539	0.017
	Time * Condition	3.641	0.069	0.137
DERS Total Score	Time	4.473	0.044	0.147
	Condition	0.167	0.686	0.006
	Time * Condition	1.359	0.254	0.050
MDI Total Score	Time	0.567	0.458	0.021
	Condition	0.035	0.854	0.001
	Time * Condition	3.164	0.087	0.105
BDI-II Total Score	Time	9.797	0.004	0.274
	Condition	0.115	0.737	0.004
	Time * Condition	0.792	0.382	0.030
BAI Total Score	Time	5.088	0.033	0.169
	Condition	0.456	0.506	0.018
	Time * Condition	0.935	0.343	0.036

*Note.* CFQ = Cognitive Failures Questionnaire; WHODAS = World Health Organization Disability Assessment Schedule 2.0; PCL-5 = PTSD Checklist for DSM-5; DERS = Difficulties in Emotion Regulation Scale; MDI = Multiscale Dissociation Inventory; BDI-II = Beck Depression Inventory II; BAI = Beck Anxiety Inventory.

**Table 6:** Results of hierarchical linear models assessing the trajectory of changes in subjective cognition and self-report symptom measures over time ^a^.

**CFQ Total Score**						
***Effect***	***b***	***SE***	***t***	***df***	***p***	***d***
Initial CFQ Severity (Intercept)	53.90	2.58	20.88	33	<0.001	
CFQ Severity Over Time (Slope)	−1.44	0.53	−2.72	33	0.010	−0.47
**PCL-5 Total Score**						
***Effect***	***b***	***SE***	***t***	***df***	***p***	***d***
Initial PCL-5 Severity (Intercept)	43.13	2.48	17.40	33	<0.001	
PCL-5 Severity Over Time (Slope)	−2.23	3.74	−5.97	33	<0.001	−1.02
**DERS Total Score**						
***Effect***	***b***	***SE***	***t***	***df***	***p***	***d***
Initial DERS Severity (Intercept)	103.02	3.67	28.11	33	<0.001	
DERS Severity Over Time (Slope)	−2.36	0.61	−3.89	33	<0.001	−0.67
**MDI Total Score**						
***Effect***	***b***	***SE***	***t***	***df***	***p***	***d***
Initial MDI Severity (Intercept)	55.95	2.71	20.64	33	<0.001	
MDI Severity Over Time (Slope)	−1.24	0.34	−3.70	33	<0.001	−0.63
**BDI-II Total Score**						
***Effect***	***b***	***SE***	***t***	***df***	***p***	***d***
Initial BDI-II Severity (Intercept)	28.07	20.2	13.90	33	<0.001	
BDI-II Severity Over Time (Slope)	−1.39	0.31	−4.56	33	<0.001	−0.78
**BAI Total Score**						
***Effect***	***b***	***SE***	***t***	***df***	***p***	***d***
Initial BAI Severity (Intercept)	25.12	2.00	12.57	33	<0.001	
BAI Severity Over Time (Slope)	−1.33	0.31	−4.25	33	<0.001	−0.73

*Note.* CFQ = Cognitive Failures Questionnaire; PCL-5 = PTSD Checklist for DSM-5; DERS = Difficulties in Emotion Regulation Scale; MDI = Multiscale Dissociation Inventory; BDI-II = Beck Depression Inventory II; BAI = Beck Anxiety Inventory. ^a^ Unit of measurement in time is one week.

**Table 7:** Results of hierarchical linear models assessing the impacts of baseline variables on the trajectory of changes in subjective cognition and self-report symptom measures over time ^a^.

**Effect of Baseline PCL-5 on CFQ Total Score Trajectory**						
***Effect***	***b***	***SE***	***t***	***df***	***p***	***d***
Initial CFQ Severity (Intercept)	48.49	2.30	21.05	27	<0.001	
PCL-5 Total	0.57	0.15	3.82	27	<0.001	0.71
CFQ Severity Over Time (Slope)	−0.97	0.59	−1.65	27	0.110	−0.31
PCL-5 Total	−0.04	−0.05	−0.92	27	0.365	−0.17
**Effect of Baseline DERS on CFQ total score trajectory**						
***Effect***	***b***	***SE***	***t***	***df***	***p***	***d***
Initial CFQ Severity (Intercept)	51.61	2.27	22.79	30	<0.001	
DERS Total	0.34	0.09	3.64	30	<0.001	0.64
CFQ Severity Over Time (Slope)	−1.25	0.56	−2.25	30	0.032	−0.40
DERS Total	−0.03	0.02	−1.69	30	0.102	−0.30
**Effect of baseline DERS on PCL-5 total score trajectory**						
***Effect***	***b***	***SE***	***t***	***df***	***p***	***d***
Initial PCL Severity (Intercept)	40.14	2.07	19.37	30	<0.001	
DERS Total	0.41	0.08	5.08	30	<0.001	0.90
PCL Severity Over Time (Slope)	−2.46	0.41	−5.98	30	<0.001	−1.06
DERS Total	0.01	0.02	0.39	30	0.702	0.07
**Effect of baseline DERS on MDI total score trajectory**						
***Effect***	***b***	***SE***	***t***	***df***	***p***	***d***
Initial MDI Severity (Intercept)	54.04	2.28	23.73	30	<0.001	
DERS Total	0.36	0.10	3.72	30	<0.001	0.66
MDI Severity Over Time (Slope)	−1.14	0.28	−4.12	30	<0.001	−0.73
DERS Total	−0.02	0.01	−2.11	30	0.044	−0.37
**Effect of baseline DERS on BDI-II total score trajectory**						
***Effect***	***b***	***SE***	***t***	***df***	***p***	***d***
Initial BDI Severity (Intercept)	25.77	1.27	20.22	30	<0.001	
DERS Total	0.36	0.05	6.80	30	<0.001	1.20
BDI Severity Over Time (Slope)	−1.47	0.30	−4.89	30	<0.001	−0.86
DERS Total	−0.00	0.01	−0.44	30	0.664	−0.08
**Effect of baseline DERS on BAI total score trajectory**						
***Effect***	***b***	***SE***	***t***	***df***	***p***	***d***
Initial BAI Severity (Intercept)	22.60	1.61	14.01	30	<0.001	
DERS Total	0.34	0.06	5.45	30	<0.001	0.96
BAI Severity Over Time (Slope)	−1.25	0.33	−3.76	30	<0.001	−0.67
DERS Total	−0.02	0.01	−1.63	30	0.113	−0.29
**Effect of baseline MDI on CFQ total score trajectory**						
***Effect***	***b***	***SE***	***t***	***df***	***p***	***d***
Initial CFQ Severity (Intercept)	51.80	1.88	27.63	31	<0.001	
MDI Total	0.62	0.16	3.88	31	<0.001	0.68
CFQ Severity Over Time (Slope)	−1.25	0.48	−2.63	31	0.013	−0.46
MDI Total	−0.06	0.04	−1.77	31	0.087	−0.31
**Effect of baseline BDI-II on CFQ total score trajectory**						
***Effect***	***b***	***SE***	***t***	***df***	***p***	***d***
Initial CFQ Severity (Intercept)	52.73	2.23	23.63	31	<0.001	
BDI Total	0.73	0.14	5.36	31	<0.001	0.93
CFQ Severity Over Time (Slope)	−1.32	0.52	−2.52	31	0.017	−0.44
BDI Total	−0.02	0.04	−0.586	31	0.562	−0.10
**Effect of baseline BAI on CFQ total score trajectory**						
***Effect***	***b***	***SE***	***t***	***df***	***p***	***d***
Initial CFQ Severity (Intercept)	52.58	1.97	26.64	31	<0.001	
BAI Total	0.87	0.14	6.34	31	<0.001	1.10
CFQ Severity Over Time (Slope)	−1.31	0.49	−2.66	31	0.012	−0.46
BAI Total	−0.08	0.04	−2.14	31	0.041	−0.37

*Note.* PCL-5 = PTSD Checklist for DSM-5; CFQ = Cognitive Failures Questionnaire; DERS = Difficulties in Emotion Regulation Scale; MDI = Multiscale Dissociation Inventory; BDI-II = Beck Depression Inventory II; BAI = Beck Anxiety Inventory. ^a^ Unit of measurement in time is one week.

## Data Availability

Data are available upon request from the authors.
